# Proteomic and functional analyses in disease models reveal CLN5 protein involvement in mitochondrial dysfunction

**DOI:** 10.1038/s41420-020-0250-y

**Published:** 2020-03-30

**Authors:** Stefano Doccini, Federica Morani, Claudia Nesti, Francesco Pezzini, Giulio Calza, Rabah Soliymani, Giovanni Signore, Silvia Rocchiccioli, Katja M. Kanninen, Mikko T. Huuskonen, Marc H. Baumann, Alessandro Simonati, Maciej M. Lalowski, Filippo M. Santorelli

**Affiliations:** 1 Molecular Medicine for Neurodegenerative and Neuromuscular Diseases Unit, IRCCS Stella Maris Foundation, Pisa, Italy; 2 Neurology (Child Neurology and Neuropathology), Department of Neuroscience, Biomedicine and Movement, University of Verona, Verona, Italy; 3 Medicum, Biochemistry/Developmental Biology and HiLIFE, Meilahti Clinical Proteomics Core Facility, University of Helsinki, Helsinki, Finland; 4 NEST, Scuola Normale Superiore, Pisa, Italy; 5 Fondazione Pisana per la Scienza, Pisa, Italy; 6 Institute of Clinical Physiology (IFC) CNR, Pisa, Italy; 7 A. I. Virtanen Institute for Molecular Sciences, University of Eastern Finland, Kuopio, Finland

**Keywords:** Proteomics, Neurological disorders

## Abstract

CLN5 disease is a rare form of late-infantile neuronal ceroid lipofuscinosis (NCL) caused by mutations in the *CLN5* gene that encodes a protein whose primary function and physiological roles remains unresolved. Emerging lines of evidence point to mitochondrial dysfunction in the onset and progression of several forms of NCL, offering new insights into putative biomarkers and shared biological processes. In this work, we employed cellular and murine models of the disease, in an effort to clarify disease pathways associated with CLN5 depletion. A mitochondria-focused quantitative proteomics approach followed by functional validations using cell biology and immunofluorescence assays revealed an impairment of mitochondrial functions in different CLN5 KO cell models and in *Cln5*^−^^/−^ cerebral cortex, which well correlated with disease progression. A visible impairment of autophagy machinery coupled with alterations of key parameters of mitophagy activation process functionally linked CLN5 protein to the process of neuronal injury. The functional link between impaired cellular respiration and activation of mitophagy pathways in the human CLN5 disease condition was corroborated by translating organelle-specific proteome findings to CLN5 patients’ fibroblasts. Our study highlights the involvement of CLN5 in activation of mitophagy and mitochondrial homeostasis offering new insights into alternative strategies towards the CLN5 disease treatment.

## Introduction

The Neuronal ceroid lipofuscinoses (NCL) are the most common inherited progressive encephalopathies of childhood characterized by epilepsy, blindness, dementia, motor impairment and premature death. Based on clinical, pathological, and molecular criteria, fourteen different forms of NCL have been described so far, associated with nearly 400 mutations (mostly autosomal recessively inherited) in 13 genes (*CLN1*-*8*, *CLN10*-*14*). The diagnosis of NCL is based on mutation analysis, but precise definition is substantiated by morphological findings, through the characteristic storage deposits which are specific for each form^1,2^. NCL specific ultrastructural patterns encompass GRODs (osmiophilic granular deposits seen in CLN1, CLN4, CLN10) and non-GROD features (curvilinear, rectilinear profiles or fingerprint profiles appearing either separate or in combination in other forms), the latter correlating with accumulation of the subunit c of ATP synthase (SCMAS)^1–3^. Currently, no treatment is available for any form of NCL, although a cohort of CLN2 patients undergoes an enzymatic replacement therapy^4,5^.

The “non-enzymatic” CLN5 disease (MIM 256731) represents a rare, late infantile form caused by mutation in the *CLN5* gene encoding a soluble, yet uncharacterized, lysosomal matrix glycoprotein which appears to be involved in endocellular trafficking at endoplasmic reticulum, Golgi and endosomes levels as well as in controlling the itinerary of lysosomal sorting receptors. In childhood, the disease exhibits a relatively slowly progressive course advancing further with visual failure, motor and mental decline, ataxia, myoclonus and epilepsy. Few postmortem pathologies are seen in CLN5 patients, as usually brains display early and pronounced atrophy in the cerebellum accompanied by storage deposition, destruction of cerebral neurons, astrocytosis and myelin loss^6^. Death usually occurs early in life, between the second and the fourth decade. A recent natural history study of the CLN5 disease highlighted the presence of two groups of patients with different clinical severity defining the conditions for experimental or disease-modifying treatments within the first 3 years of the disease^7^, as thereafter, high variability in rate of decline is evident in patients based on mutation type and residual levels of CLN5 protein.

Various CLN5 disease models have been described so far including ovine, bovine, and canine (https://www.ucl.ac.uk/ncl/animal.shtml). *Cln5* knockout (*Cln5*^−/−^) mice demonstrate a prominent homology to human pathology^8,9^, suffering from several neurological defects with relatively late onset including brain atrophy, visual/cognitive and mild motor dysfunction, and a marked glial activation and hypomyelination preceding neuronal loss, mostly pronounced in the thalamocortical system. In marked contrast to other forms of NCL, neuronal loss in this model starts in the cortex and only subsequently occurs within thalamic relay nuclei^10^. Defective myelination has instead been observed in vitro and in the developing brain, accompanied by the malfunctioning of sphingolipid transport^11^.

Mitochondrial dysfunction is commonly involved in the pathogenesis of neurodegenerative disorders since neurons highly depend on oxidative phosphorylation for their energy supply and have low capacity to upregulate glycolytic ATP generation. Defects in the mitochondrial compartment have been hypothesized in various NCL subtypes^12–16^, and appear to play relevant roles in the initiation of the apoptotic cascade, known as the basis of neuronal injury. The hypothesis of a mitochondrial dysfunction in NCL is supported by in vitro studies showing alterations of the mitochondrial network in *CLN1* and *CLN6* patients’ cells, low levels of expression of mitochondrial proteins and the implication of pathways leading to apoptosis in CLN1 disease^13^. The crosstalk of several mitochondrial carriers implicated in protein folding/sorting with CLN1, CLN3, and CLN5 proteins^14^, revealed in the interactomics studies, is marked. Changes in the level of expression of mitochondrial proteins were also observed in both thalamus and cerebral cortex of symptomatic *Ppt1*^−/−^ mice, with decreased levels of cytochrome *c* oxidase subunit 7C and two subunits part of the F_0_-ATP synthase. Furthermore, a quantitative analysis of PPT1 interaction partners in human neuroblastoma cells identified seven mitochondrial proteins including components of the pyruvate dehydrogenase and ATP synthase complexes and voltage dependent anion channel protein 2^12,17,18^. Although the precise function of many NCL causative proteins remains to be fully elucidated, the aforementioned findings point to a significant contribution of mitochondrial dysfunction in the onset and progression of the disease.

In this work, by employing quantitative mitochondrial proteomics and functional studies, we identified new disease-relevant functions of CLN5 protein and factors modifying the disease status. Mitochondrial fractions were derived from *CLN5* knockout cells and cerebral cortex in pre-symptomatic and symptomatic *Cln5*^−/−^ mice. Initially, for proof-of principle experiments, HEK 293T cells were selected given their easy handling, well suited for CRISPR/Cas9 gene editing. More robust functional analyses were subsequently performed in SH-SY5Y human neuronal-like cells, a model commonly used in studies related to neurodegenerative diseases, oxidative stress^19,20^, and other NCL forms^12,17,21^. Mitochondria-focused quantitative proteomics in *CLN5* knockout cells highlighted the mitophagy process and changes in mitochondrial function accompanied by elevation of an oxidative stress, and these altered processes were observed in the *Cln5*^−*/−*^ mouse model, already at the early stage of the disease. Finally, CLN5 patients’ skin fibroblasts were used to bridge the experimental paradigm to human pathology and to pinpoint dysregulated mitochondrial pathways.

## Results

Molecular features of *CLN5* KO cells and patients’ fibroblasts involved in this study are presented in the supplementary material.

### Analysis and bioinformatic categorization of differential mitochondrial proteome profiles

**Fig. 1:**
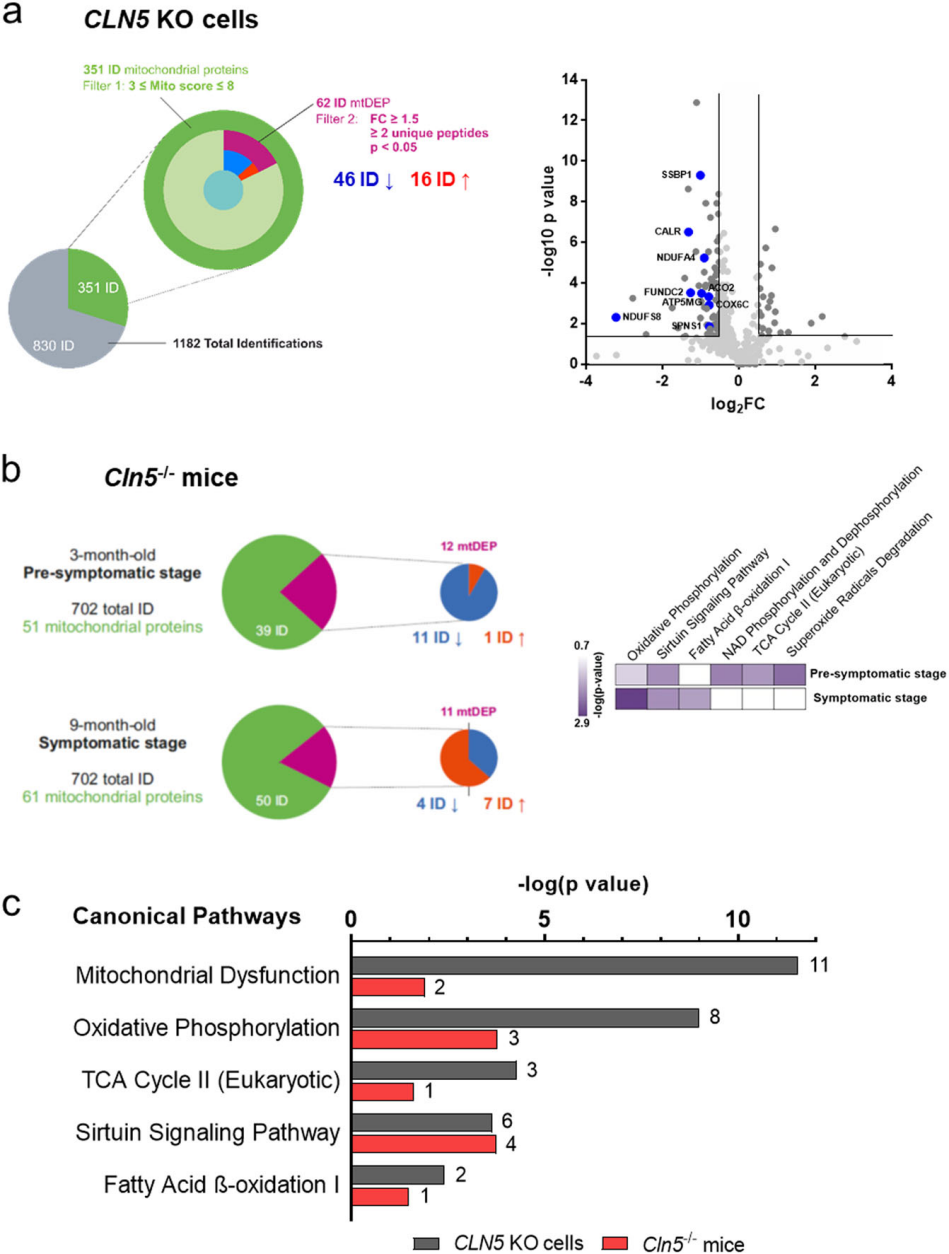
Bioinformatic examination of differential proteome profiles in analyzed disease models. **a** Left panel, nested pie chart describing the filtering strategy adopted for quantitative proteomics data in the *CLN5* knockout cell model. The number of quantified mitochondrial proteins is shown, selected based on their high and medium mitochondrial confidence (filter 1). Only these mitochondrial proteins, which passed the criterion of differential fold change ratio, FC > 1.5, based on quantitation utilizing ≥2 unique peptides and *p* value ≤ 0.05 by Anova, (mtDEPs; Filter 2) served as targets in bioinformatic surveys. Right panel, volcano plot depicting the proteomic profiles of selected mtDEPs in the cell model showing down-regulation of several mitochondrial proteins. **b** A similar filtering strategy was also applied to a *Cln5* ^*−/*−^ mouse model (FC > 1.3, ≥2 unique peptides and *p* value ≤ 0.05 by Anova). Heat map of IPA canonical pathway analysis reporting the most significantly affected pathways according to the disease progression. **c** Bar chart representation of affected canonical pathways identified in different disease models with the lowest predicted *p* values. The number of mitochondrial DEPs assigned to each category is presented.

The proteomic profiling of mitochondrial fractions from two HEK 293T KO clones (#8, #9b) was performed and the results compared to the control cells^22^. Organelle-specific proteome profiling revealed 62 mitochondrial differentially expressed proteins (mtDEPs); among these 46 mtDEPs were down-regulated and 16 up-regulated (Fig. 1a). Supplementary Table S2 lists the full dataset. Categorization of mtDEPs was performed through the use of Ingenuity Pathway Analysis (IPA)^23^. Specifically, we identified canonical pathways related to mitochondrial dysfunction and oxidative phosphorylation, whereas among the affected disease and functions, mitochondrial membrane potential, ATP synthase, mitochondrial disorder, modification of ROS, morphology of mitochondria, consumption of oxygen and metabolism of hydrogen peroxide were pinpointed among others. A molecular network encompassing 22 identified mtDEP is reported in the Supplementary Fig. S2a. Similar functional associations were also seen in the analysis based on the Gene Ontology (GO), highlighting processes related to bioenergetic metabolism, oxidative stress and protein folding (Supplementary Fig. S2b).

The mitochondrial proteome analysis was also carried out in preparations obtained from homozygous *Cln5*^−/−^ and wild-type littermates, age matched control male mice. Pre-symptomatic (3 months) and symptomatic (9 months) stages were investigated. Data filtering allowed the identification of 12 DEPs with high and medium mitochondrial confidence in presymptomatic *Cln5*^−/−^ mice (11 down-regulated and one up-regulated), while profiling experiments at the symptomatic stage revealed 11 mtDEPs, including four down-regulated and seven up-regulated ones (Fig. 1b). Comparative analysis focusing on changes in biological states across the disease progression indicated a significant impairment of oxidative phosphorylation term already at the presymptomatic stage with statistical significance increasing during the disease course (Fig. 1b). Common altered processes related to mitochondrial metabolism included NAD phosphorylation/dephosphorylation, TCA cycle and superoxide radicals degradation occurred to be significantly affected early in the pathogenesis of CLN5 disease. Common pathway elements were also identified by comparing the analyzed disease models. Both cells and mice CLN5 models indicated associations linked to dysregulation of mitochondrial function (Fig. 1c). In summary, bioinformatic surveys highlighted the functional association related to loss of CLN5 protein expression and impairment of mitochondrial compartment, thereby linking CLN5 to mitochondrial dysfunction.

### Protein expression of selected mtDEPs

**Fig. 2:**
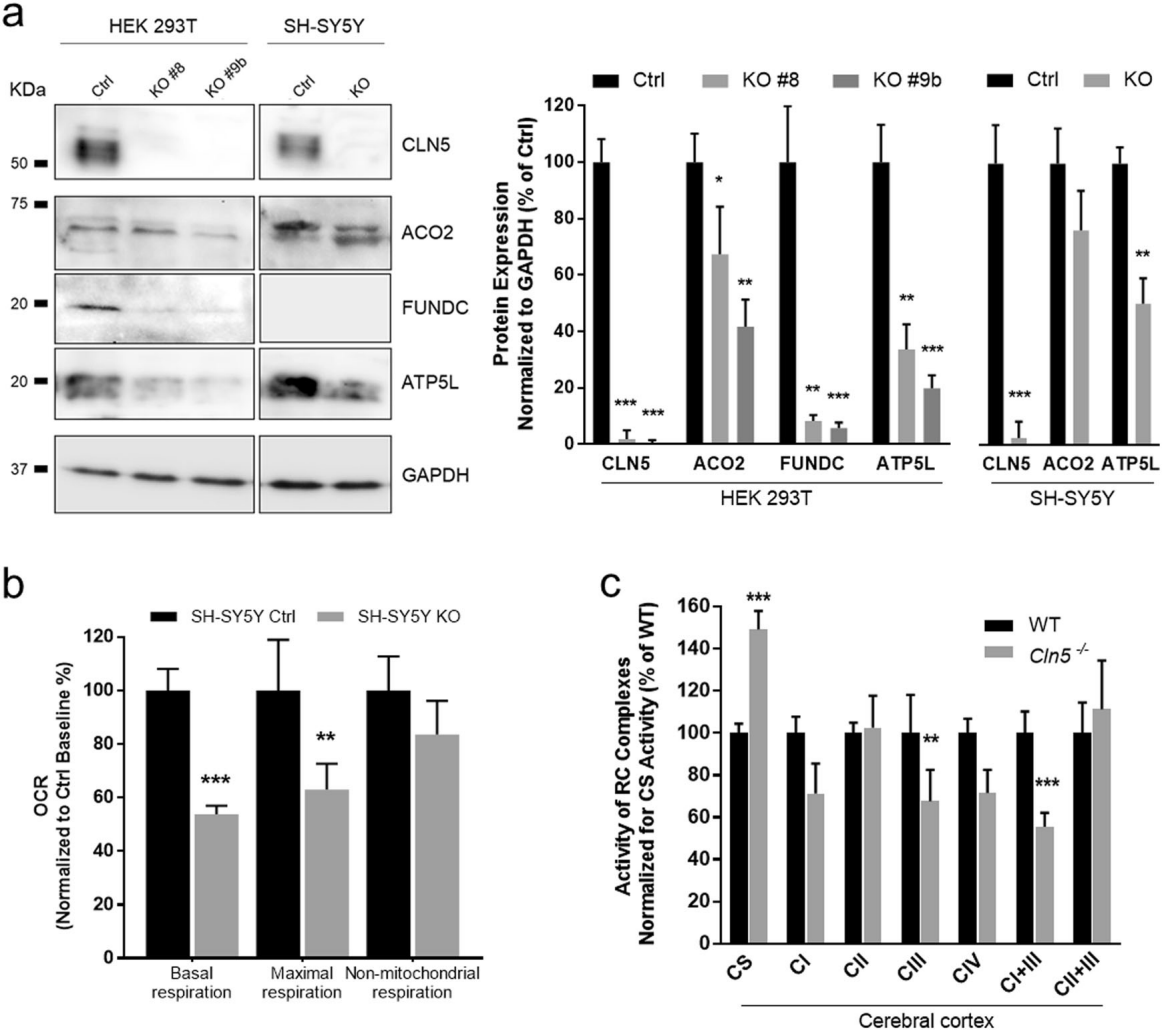
Functional and expression validation of proteomic data. **a** Validation of protein expression levels related to selected mtDEPs, assessed in HEK 293T and SH-SY5Y whole cell lysates. Densitometric analyses corroborate the results of mitochondrial proteome profiling. **b** Analysis of mitochondrial energy metabolism in neuroblastoma cells *CLN5* KO using the Seahorse XF Cell Mito Stress Test, shows a decreased basal oxygen consumption rate as well as maximal respiration whereas no significant differences were observed in non-mitochondrial respiration. **c** Spectrophotometric determination of RC enzymes in cerebral cortex from symptomatic mice reveals a general OXPHOS impairment with a significant reduction of complexes III and I + III and increase of citrate synthase activity as index of mitochondrial proliferation. Data represent the mean (± standard deviation) of three independent experiments (*n* = 9). Statistical significance was determined by two-tailed Student *t* test *p < 0.05; **p < 0.01; ***p < 0.001.

To cross validate the proteomics data with direct measurement of protein expression levels using WB, among mtDEPs detected in HEK 293T KO model, we selected proteins relevant to cellular respiration (ACO2 and ATP5L) and mitophagy process (FUNDC; Fig. 2a). Immunoblotting with specific antibodies corroborated the results of proteomic profiling demonstrating a significant downregulation of analyzed proteins in HEK 293T KO cellular lysates. Similarly, reduced levels of ACO2 and ATP5L were traced in SH-SY5Y KO cells whereas FUNDC expression was undetectable in neuroblastoma lysates.

### Analysis of cellular respiration in CLN5 models

Bioinformatic scrutiny of differential proteome profiles highlighted pathways related to mitochondrial dysfunction. In order to investigate the functional consequence of alterations in annotated proteins, we assessed micro-oxygraphy in SH-SY5Y model. Compared to the control cells, we observed a decreased basal and maximal oxygen consumption rate (OCR) without significant difference in non-mitochondrial respiration in SH-SY5Y KO cells (Fig. 2b). Even though basal rate of respiration could not adequately reflect the ability of cells to cope with an increased energy demand, the maximal respiration is a good estimate of the maximum capacity for mitochondrial substrate oxidation^24^. Furthermore, normal non-mitochondrial respiration parameter indicated that loss of CLN5 expression leads to impairment of cellular respiration in KO cells. Spectrophotometric determination of RC enzymes activity in cerebral cortex from symptomatic *Cln5*^*−/*−^ mice revealed multiple defects with significant reduction of complexes III and I + III and an increase of citrate synthase (CS) activity, a validated index of mitochondrial mass (Fig. 2c). In contrast, no significant differences were measured in heart tissue from affected mice, an organ that is not involved in the pathogenesis of CLN5 disease (data not shown).

### Analysis of mitophagy in CLN5 models

#### Qualitative analysis of mitochondrial network and measurements of mitochondrial membrane potential

**Fig. 3:**
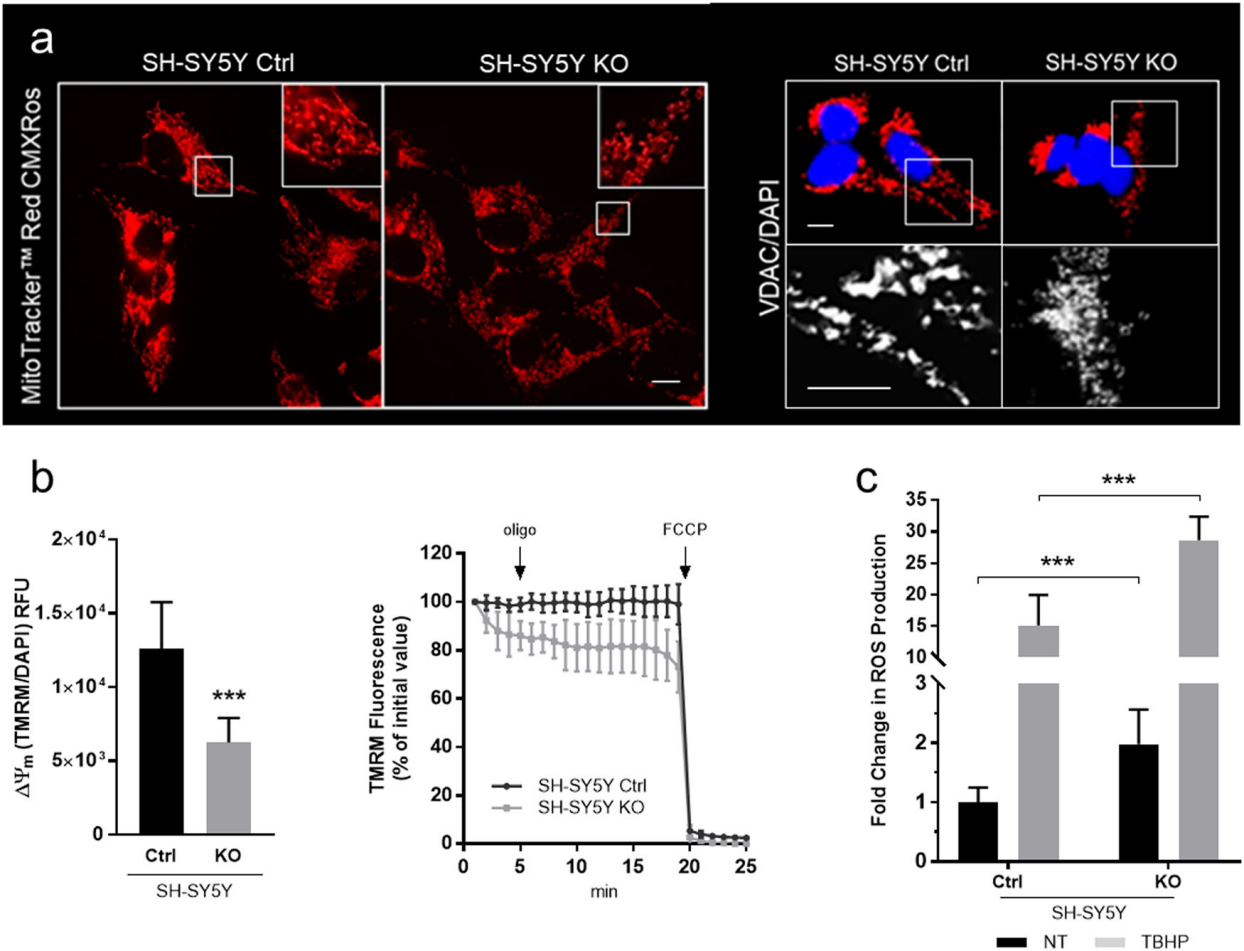
Analysis of the mitophagy-related parameters in CLN5 cell model. **a** Mitochondrial network organization in SH-SY5Y KO model. When compared to the empty vector control line, *CLN5* KO cells reveal an altered mitochondrial network with increased fragmentation both after Mitotracker red and VDAC staining. MT red CMXRos probe accumulation, which is dependent upon membrane potential, is reduced in KO lines suggesting a decreased ΔΨm. Figure shows representative images from three independent cell staining. Scale bar, 10 µm. Inserts show a 3x magnification. **b** Neuroblastoma cells were stained with TMRM probe revealing in *CLN5* KO line a reduced mitochondrial membrane potential both in terms of probe accumulation and membrane potential maintenance. End-point assay indicates a mitochondrial membrane depolarization in KO cells reported as average TMRM relative fluorescence units RFU ± SD subtracting the fluorescence related to FCCP treatment. Data were normalized by DAPI staining as a function of cell number. Kinetic track demonstrates a differential ability between KO and control cells to maintain polarized the mitochondrial membrane particularly after oligomycin blocking proton transit through Complex V, highlighting any leakage of inner mitochondrial membrane. FCCP was added at the end of the experiments to fully depolarized mitochondrial to demonstrate specificity of the acquired measurements. **c** Redox state of cells lacking CLN5 using the fluorogenic dye H2DCFDA shows a significantly larger amount of ROS in SH-SY5Y *CLN5* KO cells as compared to controls both in regular medium (RM) and under stress condition (short-term TBHP treatment). Data represent the mean (± standard deviation) of three independent experiments (*n* = 9). Asterisks indicate statistical significance of Ctrl versus KO cells in the presence/absence of TBHP treatment, as determined by the Student *t* test. ***p < 0.001.

To investigate how impaired cellular respiration related to CLN5 deficiency impacts on mitochondrial structure, we imaged the shape of the mitochondrial reticulum in SH-SY5Y KO cells. When compared to the empty vector control line, the staining of neuroblastoma KO with MitoTracker Red CMXRos dye and anti-VDAC1 monoclonal antibody (two mitochondrial markers) revealed a fragmented mitochondrial network with an altered distribution around the nuclei (Fig. 3a), as previously observed in primary cells from cases with CLN1 disease^13^. Moreover, chloromethyl-X-rosamine (CMXRos) accumulation, which is dependent on membrane potential, was reduced in SH-SY5Y KO cells suggesting a decrease in the mitochondrial membrane potential (ΔΨm). Reduction of ΔΨm was further assessed by staining the neuroblastoma cells with TMRM probe revealing a reduced mitochondrial membrane potential in KO cells in terms of both probe accumulation and membrane potential maintenance. Furthermore, in KO cells treatment with oligomycin, which blocks proton transit through CV, highlighted an increased leakage of the inner mitochondrial membrane independently of ATP synthesis (Fig. 3b). In conclusion, ΔΨm is a key component in the mitophagy pathway and the reduction of this parameter or its dissipation over time involves mainly non-fusing mitochondria, and is closely related to the depolarization events induced by ROS overproduction^25,26^.

#### Evaluation of the redox state in CLN5 KO SH-SY5Y cells

Due to their role in metabolism, mitochondria are very susceptible to oxidative stress and formation of ROS as a mitochondrial waste product eventually leads to cytotoxicity and cell death. To verify the redox state of cells lacking CLN5, hydroxyl, peroxyl and other reactive oxygen species were investigated using the cell permeant fluorogenic dye H2DCFDA. Both in regular medium (RM) and under stress conditions (short-term treatment with TBHP as a source of free radicals), we observed a significantly increased formation of ROS in KO versus controls SH-SY5Y cells, indicating an increased susceptibility to oxidative stress (Fig. 3c).

#### Monitoring mitophagy through the expression of mitochondrial probes and markers of the autophagy machinery

**Fig. 4:**
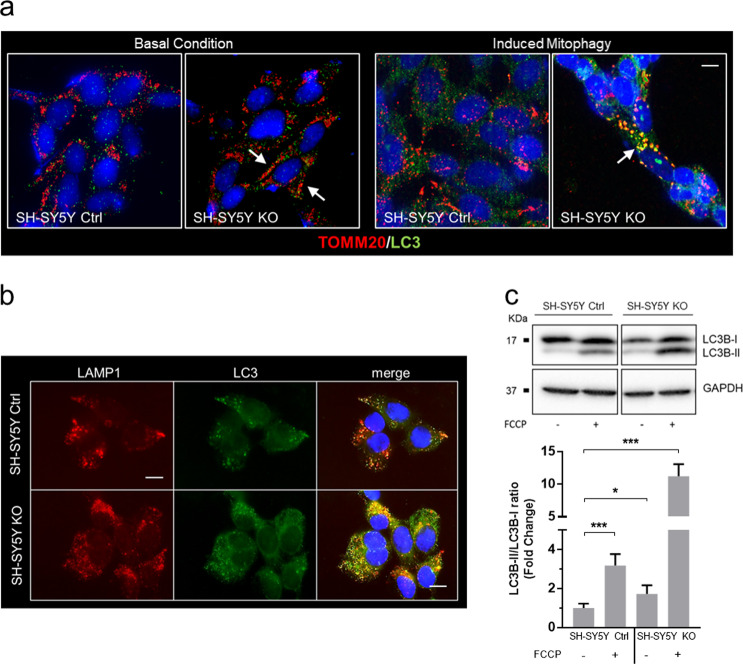
Dysregulation of autophagy and mitophagy machinery in CLN5 disease. **a** Colocalization of mitochondria (marked by TOMM20) and autophagosomes (marked by LC3) was markedly increased in SH-SY5Y *CLN5* KO cells (arrows, yellow fluorescence), and strongly associated with FCCP treatment to stimulate mitophagy (right panel, 20 µM FCCP). Scale bar, 10 µm. **b** An increase in co-localization of LC3 (in green) and LAMP1 (in red) marked by yellow fluorescence suggested a compromising cargo degradation in SH-SY5Y *CLN5* KO cells with dysregulated autophagosome-lysosome fusion. DAPI (blue fluorescence) was used to stain nuclei. Scale bar, 10 µm. **a**, **b** show representative images from three independent cell staining. **c** Western blotting analysis of the autophagy marker LC3 in SH-SY5Y cells indicates a significant reduction in the level of the autophagic flux (LC3BII/LC3BI ratio), exacerbated by the addition of 20 µM FCCP. Data represent the mean (± standard deviation) of three independent experiments (*n* = 9). Student *t* test. ns not statistically significant; *p < 0.05; **p < 0.01; ***p < 0.001.

In mitophagy, mitochondrial fragmentation is closely linked to autophagosome formation. In KO cells, using TOMM20 and LC3 as markers of mitochondria and autophagosomes, respectively, we observed distinct co-localization of both markers, particularly evident under FCCP treatment, suggestive for an activated mitophagy process (Fig. 4a). Both ATP depletion and oxidative stress contribute to activation of the stress-induced mitophagy pathways, and damaged mitochondria tend to fragment^27^. This alteration in mitochondrial dynamics occurs simultaneously with autophagosome formation, a pathway known to be implicated in NCL, including CLN5^28,29^. In SH-SY5Y KO cells we further demonstrated an autophagosome accumulation by double staining with LAMP1 (lysosomal marker) and LC3 (autophagosome marker; Fig. 4b), and a significant increase in LC3-II/LC3-I ratio, both in basal condition and after mitophagy activation with 20 µM FCCP for 2 h (Fig. 4c).

### Relevance to human CLN5 disease

**Fig. 5:**
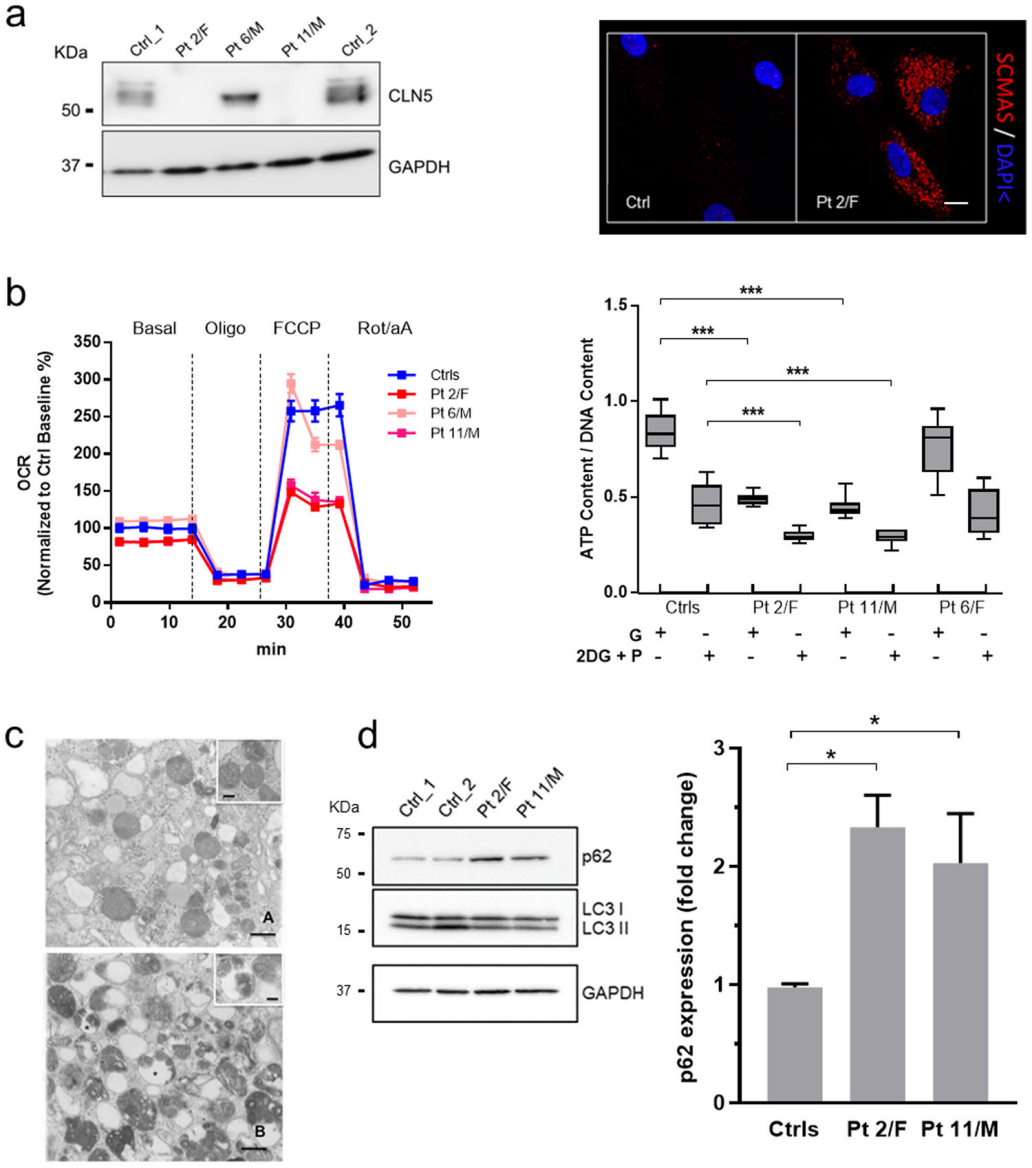
Translational confirmations in patient’ skin fibroblasts. **a** CLN5 protein levels, measured by Western Blotting, show a full lacked CLN5-immunoreactivity in Pt 2/F and 11/M, whereas a different CLN5 expression pattern is shown in patient 6/M, likely due to different effects of *CLN5* mutations exerted on protein synthesis. Immunochemical studies demonstrate that subunit c of mitochondrial ATP synthase (SCMAS), the characteristic hallmark in NCL5, is stored in intracellular aggregates in patients’ fibroblasts. Scale bar, 10 µm. **b** Micro-oxygraphy track shows a reduced OCR, which is clearly evident after FCCP injection, reflecting a deficient spare respiratory capacity. Moreover, under oxidative conditions (blocking glycolysis with 2-deoxy-d-glucose + pyruvate) a reduced ATP content and bioenergetics defects in patients with more severe mutations and less abundant protein levels are seen. G glucose used as source for ATP production; O oligomycin, used to block mitochondrial respiration; 2DG + P = 2-deoxy-d-glucose plus pyruvate, used to block glycolysis. **c** Fibroblast pellets were fixed with 1.25% glutaraldehyde and 0.5% paraformaldehyde in phosphate buffer, post-fixed in 1% osmium tetraoxide and stained with uranyl acetate and lead citrate. Abnormal cytoplasmic pattern is marked, as demonstrated by the increased amount of vacuoles, dense bodies and lysosomes (A and B). Osmiophilic figures with different ultrastructural arrangement, including honeycomb structures can be observed (insert A), but classical cytosomes are not detectable. Several vacuoles, outlined by a single membrane and containing osmiophilic material, are consistent with features of autophagic process. Osmiophilic inclusions featuring multilamellar structures can be detected within the vacuoles (insert B). Scale bar = 1.0 µm; insert A, scale bar = 0.2 µm; insert B, scale bar = 0.3 µm. **d** Western Blotting analysis reveals significantly increased expression of p62 in patients’ fibroblasts, consistent with a block of autophagosome-lysosome maturation. Data represent the mean (± standard deviation) of three independent experiments (*n* = 9). Statistical significance was determined by the Student *t* test. *p < 0.05; **p < 0.01; ***p < 0.001.

To rapidly assess whether the mitochondrial changes seen upon proteome profiling and functional studies in KO cells and mice were relevant to the CLN5 disease, we selected three fibroblast primary lines with biallelic mutations in the *CLN5* gene and the reduced expression of CLN5 protein^7^. Two patients (2/F and 11/M) with a severe reduction of CLN5 protein (Fig. 5a) showed a dramatic reduction of basal and maximal OCR (Fig. 5b) and an increased expression of p62 (Fig. 5c), suggestive for a block of autophagosome-lysosome maturation^30^. Correspondingly, ultrastructural investigations of patients’ fibroblasts in vitro showed increased amounts of both lysosomes and vacuoles containing densely packed, osmiophilic material (Fig. 5d). Taken together, the mitochondrial changes observed in disease models were successfully replicated in CLN5 patients’ cells.

## Discussion

This study illustrates a functional proteomics strategy defining a new mitochondrial role of CLN5 protein in disease models and primary cells from NCL patients. We used KO neuronal-like cells and brain tissues from a published *Cln5*^*−/*−^ mice strain^9^ and utilized label-free quantitative proteomics targeting mitochondria, thereby reducing the complexity of samples. Identification of DEPs specifically linked to mitochondrion resulted in focused functional assessments, specifically pinpointing lowered levels of oxygen consumption and ATP production in KO cells, multiple defects in RC enzyme activities in *Cln5*^−*/−*^ mice, abnormal organization of the mitochondrial network and increased ROS production^31,32^.

These results implicate CLN5 in mitochondrial function. Along with other NCL proteins, CLN5 is a known partner of the F_1_ subunit c of ATP synthase, essential for mitochondrial ATP synthesis^33^ and interacts with mitochondrial carriers involved in the protein folding/sorting proteins, some of those are shared with other NCL proteins (i.e. CLN1 and CLN3^14^). Our findings propose CLN5 as a new player in the complex dynamics of mitochondria and potentially relevant to mitochondrial fusion/fission. Mitochondrial shape is closely linked to the function since morphological adaptations are crucial for many physiological processes such as cell cycle, immunity, apoptosis and mitochondrial quality control^34^. A damaged network has been reported in animal models for NCL^15,16^, and more recently, different patterns and degree of disorganization of mitochondrial cristae have been reported in iPSCs obtained from reprogramming CLN3 patients’ fibroblasts, cerebellar precursor cells in a Cln3Δed7/8 and primary cultures from *Cln3*^−/−^ neurons^35–37^. Furthermore, oxidative phosphorylation is critical especially for neurons, in which the compensatory capacity of the glycolytic pathway to generate ATP is particularly ineffective. In this scenario, CLN5 seems to drive the appropriate mitochondrial shape, function, and distribution within the cell, a role not previously envisaged. This would prompt enhanced oxidative stress and bioenergetic failure, the abnormalities already seen in lysosomal storage disorders including several forms of NCL^38–40^.

The combination of protein molecular signatures and immunofluorescence validation in neuronal-like KO cells suggests mitophagy as a novel aspect in the pathogenesis of CLN5 disease. The reduction of ΔΨm triggers the mitophagy pathway that induces autophagosome formation around defective mitochondria. This feature is supported by the observed dysregulation in expression of FUN14 domain-containing protein (FUNDC) (Fig. 2a, b), which is believed to serve as mitophagy receptor in mammals, and SQSTM1/p62 (Fig. 5d), thereby linking mitochondria with the autophagy mechanism in different cell types^41^. Moreover, SH-SY5Y KO cells demonstrated compromising cargo degradation combined with increased co-localization of mitochondria and autophagosome markers TOMM20 and LC3 (Fig. 4a), suggestive for mitophagy activated process. By scrutinizing GO biology and pathway data with results of functional mitochondrial assessments in this work, it seems plausible to speculate that impaired mitophagy represents a possible inducer of the process of neuronal injury, associated with reduced synapse formation and impaired neuritogenesis. It is noteworthy that severe axonal degeneration and increased neurogenesis have been demonstrated in *Cln5*-deficient mice^42^. Mitochondrial dysfunction and defective mitophagy have also been associated with more common neurodegenerative diseases including Parkinson’s disease and Frontotemporal dementia^43–45^, and the precise characterization on these associated phenomena could likely offer new opportunities for molecular targets in NCL therapy.

In this work, we demonstrate that the aforementioned mitochondrial roles and mitophagy are relevant to CLN5 disease and the cortical brain involvement seen in NCL. Contrary to heart tissue, murine cerebral cortices showed a trend towards downregulation in expression of mitochondrial proteins and related-dysregulated processes (Fig. 1b), already at the pre-symptomatic stage (age 3 months).

**Fig. 6:**
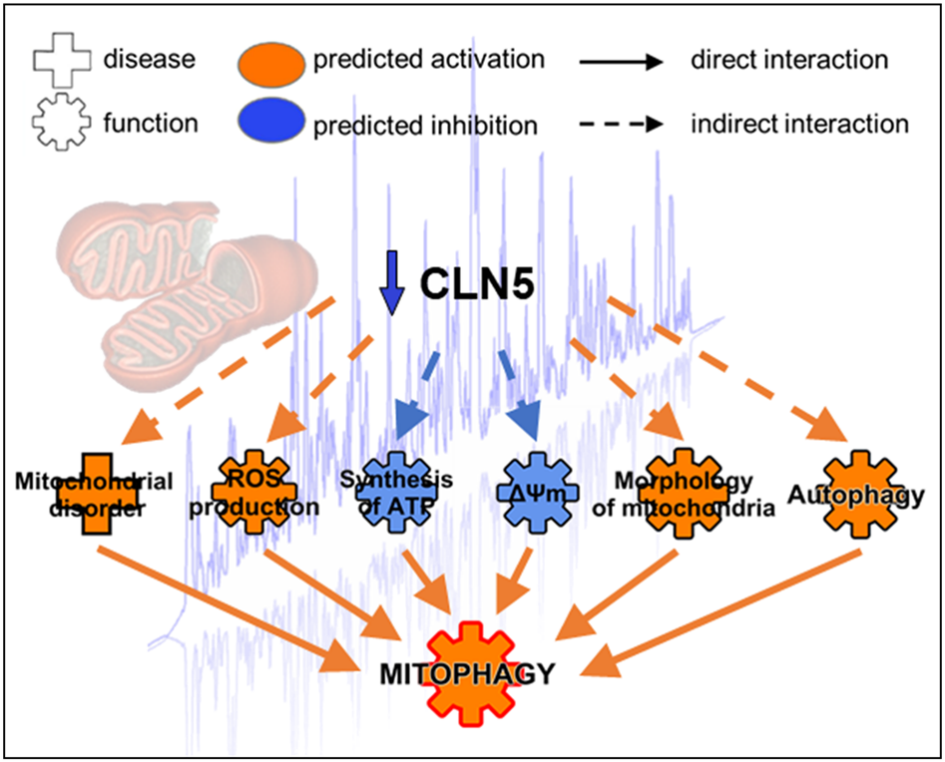
Schematic diagram of the identified processes underlying CLN5 disease models and patient material. Functional studies corroborate the bioinformatic analyses of differentially expressed mitochondrial proteins linking the lack of CLN5 with oxidative stress, bioenergetic impairment, and autophagy induction resulting in an activation of mitophagy process.

An implication of mitochondrial dysfunction and TCA cycle II has previously been reported in a proteomic study of pre-symptomatic thalami in *Ppt1*^−/−^ mice^12^. Moreover, comparing our dataset with ones generated by previous proteomic studies^12,14,46^, a considerable overlap between mtDEPs identified in *Ppt1*^*−/*−^, *Tpp1*^*−/−*^ mouse studies, and CLN3/CLN5 interaction partners has been identified (Supplementary Table S4). Shared mitochondrial carriers (MDH2, PITRM1, SLC25A3, SPNS1), proteins implicated in apoptotic (CARL, CLIC4, YWHAQ) and metabolic processes (AK2, DLST, ECI1, HKDC1, GANAB), astrocyte development (VIM), mitochondrial organization (CHCHD3, VAT1) and energy production (NDUFV2, ENO1) further reinforces the hypothesis of a common molecular theme^14,47,48^ and neuronal impairment in NCLs (Fig. 6). Furthermore, we noticed multiple defects in RC enzyme complexes (I + III and III) in cerebral cortex (Fig. 2c). Decreased activities of respiratory chain complexes have already been recognized in various forms of NCL^15,16,49^ including CLN1^13^, but it is unclear if this is a consequence of compromised fission/fusion metabolism or it is linked to other proteolytic impairments^50^. Importantly, primary cells from CLN5 patients recapitulated the findings observed in CLN5 models (i.e., reduced oxidative metabolism and elevated ROS production) in relation to the disease status and protein residual activity indicating that dysfunction in mitochondrial energy homeostasis is relevant to human disease and its progression.

The proteomic approach utilizing compartment targeted, label-free quantitative mass spectrometry is well suited to reveal specific changes and characterize disease-associated functional modules. The possibility to analyze affected animals at different disease progression stage, allowed us to identify and compare common molecular processes related to progression or shared with other NCL forms. In our example, functional proteomics revealed mitochondrial involvement in disease pathogenesis, which might serve to define new putative targets of therapeutic interest in CLN5 disease. The specific information about the role of autophagy and impaired mitochondrial function could have an impact on pathogenetic processes involved in neuronal injury and on the broad meaning of “lysosomal” pathogenesis hypothesized in more frequent neurodegenerative diseases leading to dementia in children and adults^3,51^.

## Material and methods

### Cell cultures

HEK 293T cell line (293T ATCC® CRL-3216™), derived CRISPR KO (hereafter called KO) and empty-vector px459 expressing cells (hereafter named as Ctrl) were cultured in DMEM containing 10% FBS, 4.5 g/L glucose and 1% penicillin/streptomycin, supplemented with l-glutamine to achieve a final concentration of 6 mM.

Neuroblastoma cell line (SH-SY5Y ATCC® CRL-2266) and derived KO and Ctrl clones were cultured in MEM/F12 1:1 with 10% FBS, 2 mM l-glutamine, 1% penicillin/streptomycin. Human fibroblasts were collected according to standard procedures from diagnostic skin biopsies. Patients’ parents signed an informed consent form for skin biopsy, authorizing research purposes in accordance with our Tuscany Region Ethic committee. Primary fibroblast cell lines from CLN5 patients carrying different mutations in *CLN5* have been described before^7^, and were grown at 37 °C with 5% CO_2_ in Dulbecco’s modified Eagle’s medium (DMEM), containing 10% fetal bovine serum (FBS), 4.5 g/L glucose and 1% antibiotics/antimycotics. All cell lines have been tested for mycoplasma contamination.

### *Cln5*^*−/*−^ mouse model and tissue collection

The CLN5 mouse model of late infantile Finnish variant of NCL was originally generated at the University of Helsinki^9^, by disrupting exon 3 of a mouse *Cln5* gene, with a resulting premature stop codon in exon 4 of the murine gene. Animals involved in this study were made available by the A.I. Virtanen Institute for Molecular Sciences, University of Eastern Finland, Kuopio. Both male and female mice at pre-symptomatic (3 months) and symptomatic stages (9 months) were used. Age matched wild-type siblings served as controls. A complete list of mice employed in this study is reported in Supplementary Table S1. All mice were maintained on the C57BL/6JRccHsd background. Animals were group-housed in standard conditions of maintenance^52^. Food and water were provided ad libitum. The mice were terminally anesthetized with tribromoethanol (Avertin, Sigma-Aldrich, St. Louis, MO) followed by decapitation without transcardial perfusion. For each set of mice, cerebral cortex area was isolated, weighed and maintained on ice until isolation of mitochondrial fractions. Fresh-frozen heart and cerebral cortex samples were also collected to assess Respiratory Chain (RC) complex enzyme assays. All experiments were approved by the national Animal Experiment Board of Finland and followed the animal protection guidelines of the Council of the European Union.

### Generation of *CLN5* KO cell lines using CRISPR/Cas9 system

The *CLN5* CRISPR single guide RNA (sgRNA) sequence *CATGCGCCGGAACCTGCGCT* was designed in order to efficiently target the *CLN5* gene with a minimal risk of off-target^53^, and the annealed guide oligoduplex was placed inside the pSpCas9(BB)-2A-Puro V2.0 plasmid (Addgene Plasmid #62988) backbone generating the plasmid expression vectors used in this study. Construct was then transformed into a competent *E. coli* strain (One Shot Stbl3 Chemically Competent *E. coli*; Invitrogen-ThermoFisher Scientific, Waltham, MA), and the plasmid DNA was isolated (QIAprep® spin miniprep Kit; Qiagen, Hilden, Germany) to check for the correct insertion of CRISPR guide.

HEK 293T and SH-SY5Y cell lines were transfected by Lipofectamine 3000 (Invitrogen-Thermo Fisher Scientific, Waltham, MA) following the standard protocol, and the transfection pool was puromycin selected (2 or 5 μg/mL for HEK 293T and SH-SY5Y, respectively) prior to clone isolation by “limiting-dilution technique”.

Clones grown under regular conditions (growing-time ≅ 3 weeks) were screened by Western blotting (WB) analysis, and clones lacking CLN5-immunoreactivity were evaluated by standard sequencing methods. Tracking of Indels by Decomposition (TIDE) web tool (https://tide-calculator.nki.nl), was used to accurately characterize and quantify the induced genome editing events^54^. Two HEK 293T and one SH-SY5Y *CLN5* stable KO clones (editing efficiency > 80%) were selected for downstream analyses (Supplementary Fig. S1a, b).

### Oxygen consumption rate measurements

Oxygen consumption rate (OCR) was measured in KO cell models and CLN5 cultured fibroblasts (together with their respective controls), using an XFe24 Extracellular Flux Analyzer (Seahorse Bioscience, Agilent, Santa Clara, CA). Cells were plated in XF 24-well cell culture microplates at a density of 6E + 04 cells/well and 5E + 04 cells/well for cell lines and primary fibroblasts, respectively. Measurements of endogenous respiration were performed with non-buffered DMEM medium supplemented with 1 mM pyruvate, 2 mM glutamine and 10 mM glucose. After baseline measurements, OCR was analyzed by the sequential injection of 1 mM of oligomycin, 2 mM of carbonyl-cyanide 4-(trifluoromethoxy)phenylhydrazone (FCCP) and 0.5 mM of rotenone plus antimycin A (all chemical were from Sigma Aldrich, St. Louis, MO). Data were expressed as pmol of O_2_/min normalized post-assay by the fluorescence CyQUANT Cell Proliferation Assays (Invitrogen™, Carlsbad, CA), as reported elsewhere^55^.

### Determination of reactive oxygen species

For the evaluation of intracellular reactive oxygen species (ROS) production, a cellular ROS detection assay kit (Abcam, Cambridge, UK) was used. Skin fibroblasts were labeled with the oxidative stress marker 2′,7′-dichloro-dihydrofluorescein diacetate (H2DCFDA), 25 μM for 45 min at 37 °C and then cultured for an additional hour in the presence/absence of 150 µM tert-butyl hydrogen peroxide (TBHP), a ROS-mimic compound. Cells were then analyzed on a SpectraMax® ID3 plate reader (Molecular Devices, San Jose, CA) at a wavelengths Ex/Em: 485/535 nm, and the difference in ROS levels between treated and untreated condition were expressed as relative fluorescent units (RFU) after background subtraction. For each well DCF signal was normalized to Hoechst 33342 intensity.

### Respiratory chain enzyme analysis in mouse tissues

The enzymatic activities of RC complexes were assayed spectrophotometrically in fresh-frozen hearts and cerebral cortex from symptomatic mice comparing with age matched wild-type littermates. Samples were homogenized in sucrose homogenization buffer (250 mM sucrose, 20 mM Tris-HCl, 40 mM KCl, 2 mM EGTA, pH 7,4), and protein concentration was determined by BCA assay method. RC kinetic assays were performed at 30 °C using a Beckman Coulter DU760 (Beckman Coulter, Pasadena, CA) spectrophotometer following standard methods already reported^56^. Briefly, complex I (NADH:ubiquinone reductase) activity was measured by the rotenone sensitive oxidation of NADH at 340 nm, while complex II (succinate dehydrogenase) activity was measured by the malonate sensitive reduction of succinate at 600 nm. Complex I/III (NADH:cytochrome *c* reductase) activity was measured by NADH dependent of cytochrome *c* at 550 nm, followed by complex II/III (succinate:cytochrome *c* reductase) activity measured by succinate dependent reduction of cytochrome c at 550 nm. Complex IV (cytochrome *c* oxidase activity) was assessed by the oxidation of reduced cytochrome *c* at 550 nm. The enzymatic specific activities for each mitochondrial enzyme were calculated as nmol/min/mg of protein and expressed as ratios in relation to the activity of citrate synthase (CS), a mitochondrial matrix enzyme, and determined by the formation of 5-thio-2-nitrobenzoic acid at 412 nm.

### Evaluation of mitochondrial membrane potential (ΔΨm)

Neuroblastoma cell lines were plated at 5E + 04 cells/well density in 96 well plates with normal growth medium. Following 24 h of growth, the mitochondrial membrane potential was measured using the fluorescent dye tetramethylrhodamine methyl ester (TMRM, Invitrogen™, Carlsbad, CA). The dye was loaded into cells in 100 nM in bicarbonate and phenol red-free Hank’s balanced salt solution (HBSS) supplemented with 10 mM HEPES (Sigma-Aldrich), 2 µM cyclosporine-H (CsH), pH 7.4 and placed at 37 °C for 5 min. Fluorescence was measured on a SpectraMax® ID3 plate reader (Molecular Devices, San Jose, CA) (544/590 nm Ex/Em, bottom reading). Assay was performed in parallel as described above with addition of 20 μM FCCP, which collapses the mitochondrial membrane potential. All data were expressed as the total TMRM relative fluorescence units (RFU) minus the FCCP treated TMRM fluorescence and normalized to the number of cells using 4′,6-diamidino-2-phenylindole (DAPI) staining (358/461 nm Ex/Em, bottom reading). Kinetic evaluation of ΔΨm was performed by live imaging as previously reported^57^. Briefly, neuroblastoma cell lines were seeded at 60% confluence on 35-mm glass bottom dishes (WillCo Wells B.V., Amsterdam, The Netherlands) and grown for two days in DMEM. Cells were incubated in bicarbonate and phenol red-free HBSS, supplemented with 10 mM HEPES (Sigma-Aldrich) and 1.6 µM CsH and loaded with 20 nM TMRM for 30 min at 37 ˚C. Cellular fluorescence images were acquired every minute using a Nikon Ti2-E inverted microscope equipped with a DS-Qi2Mc camera and collected with a Nikon ×60 Plan Apocr λ (NA = 1.40) oil immersion objective, using a TRITC filter set. After 5 min of baseline, oligomycin (2.5 µM final concentration) was added to the media recording sequential digital images for 15 min. At the end of each experiment, mitochondria were fully depolarized by the addition of 4 µM FCCP. Clusters of mitochondria (15–30 on average) were identified as regions of interest (ROIs), and fields without cells were used as a background. In all sequential digital images and for each ROI, the changes in fluorescence intensity were measured using Image J software (https://imagej.nih.gov/ij/)^58^. Fluorescence values were expressed as a percentage of baseline (*T*_0_, 100%), and reported as average of ROIs ± SD for each time point.

### Proteome analyses

#### Cellular fractionation method for mitochondria

Isolation of mitochondrial fractions from HEK 293T cell lysates and mice cerebral cortex was performed using Qproteome Mitochondria Isolation Kit (Qiagen, Hilden, Germany) according to manufacturer instructions. A pellet from about 1E + 07 HEK 293T cells (harvested without trypsin) or 40 mg of fresh cerebral cortex homogenized tissue were processed to obtain high-purity mitochondrial preparations. Solubilization of organelle fractions was carried out in a lysis buffer containing 7 M urea, 2 M thiourea, 4% CHAPS and protease inhibitors. The protein concentration was determined using a colorimetric assay based on the Bradford method (Bio-Rad Laboratories, Inc., Hercules, CA).

#### Sample preparation, proteolytic digestion and DIA-HDMS^E^

Ten µg of total protein from mitochondrial fraction obtained from either HEK 293T cell lysate or mice cerebral cortex were digested using a modified FASP protocol as described^17^. Three-hundred nanograms of digested proteins (three technical replicates per sample) were used for DIA (Data Independent Acquisition) HDMS^E^ (High Definition Mass Spectrometry) analysis. The parameters of DIA-HDMS^E^ runs were described previously^58,59^. Database searches were carried out against human (release 2017_48461 entries) or *Mus musculus* (release 2017_16869 entries) UniProtKB–Swiss-Prot, reviewed, database with ion accounting algorithm and using the following parameters: peptide and fragment tolerance: automatic, maximum protein mass: 500 kDa, minimum fragment ions matches per protein 7, fragment ions matches per peptide 3, peptide matches per protein 1, primary digest reagent: trypsin, missed cleavages allowed: 2, fixed modification: carbamidomethylation C, variable modifications: NQ deamidation, oxidation of methionine (M) and false discovery rate (FDR) below 4%. Protein quantitation was performed entirely on non-conflicting protein identifications, using precursor ion intensity data and standardized expression profiles. The proteomics data were submitted to MassIVE (accession number MSV000084880; 10.25345/C5PQ4G) and ProteomeXchange (PXD017356).

#### Development of a scoring system for mitochondrial proteins

Additional filters were applied to final data set in order to increase the stringency of accepted protein leads. Protein identifiers (IDs) obtained in HDMS^E^ analysis from mitochondrial fractions were selected generating a ranking for final score of mitochondrial confidence using MitoMiner 4.0 v2018 JUN (http://mitominer.mrc-mbu.cam.ac.uk), based on individual scores from various mitochondrial and functional annotation databases, and following a modified scheme from Laakkonen et al.^59^. Criteria employed in the scoring system for mitochondrial confidence are reported in Supplementary Table S3. Identifiers with a total score of 6–8 were selected as mitochondrial proteins of High Confidence (Mito HC dataset) whereas IDs with a score 3 and 5, were selected as hits with Medium Confidence (Mito MC dataset). IDs with a score below 3 were discarded from the data set. The number of unique peptides used for label-free quantitation ≥2 further filtered all data sets; and fold change (FC) from averaged, normalized protein intensities ≥1.3, for dataset from mice tissue and ≥1.5 for HEK 293T cell line, in either direction of up- or down-regulation and *p* ≤ 0.05 by ANOVA for all comparisons.

### Immunofluorescence staining

The cells adherent on sterile glass coverslips, previously treated with Poly-D-Lysine (Sigma-Aldrich) were fixed in cold methanol for 20 min and permeabilized with 0.1% Triton X-100 in PBS1X for 15 min. Coverslips were washed in PBS and incubated in a blocking solution (FBS 20% in PBS) for 1 h at room temperature. Overnight incubation at 4 °C was performed with the following primary antibodies: mouse monoclonal anti-Porin (MitoScience; dilution 1:500), mouse monoclonal anti-p62 (BD Transduction Laboratories^TM^; dilution 1:200), rabbit polyclonal anti-TOMM20 (Santa Cruz Biotechnology Inc.; dilution 1:100), mouse monoclonal anti-Human LAMP1 (BD Pharmingen^TM^; dilution 1:1000) and rabbit polyclonal anti-LC3B (Sigma-Aldrich; dilution 1:1000). As secondary antibodies, goat anti-mouse or anti-rabbit antibodies conjugated with AlexaFluor 488 or AlexaFluor 555 dye (Cell Signaling Technology Inc; dilution 1:1000) were used for 1 h at room temperature in a humid chamber. Nuclei were stained with the fluorescent dye 4,6-diamidino- 2-phenylindole-dihydrochloride (5 µg/ml DAPI, Sigma-Aldrich). Stained cells were mounted for microscopy. Images were acquired using a Nikon Ti2-E inverted microscope equipped with a DS-Qi2Mc camera and collected with a Nikon ×60 Plan Apocr λ (NA = 1.40) oil immersion objective, using a FITC, TRITC and DAPI filter sets. Mitochondrial staining with MitoTracker Red (Invitrogen-ThermoFisher Scientific), was performed using a working concentration of 200 nM in regular medium for 30 min. Following the incubation period, the cells were washed twice in PBS prior to live imaging.

### Western blotting

For Western blotting, samples were homogenized in RIPA buffer (150 mM NaCl, 50 mM Tris-HCl, 6 mM EDTA, 1% NP-40, 0.1% SDS, 0.5% deoxycholic acid, pH 8.0) containing inhibitors of proteases (Roche Diagnostics GmbH, Mannheim, Germany) and centrifuged for 10 min at 14000*×g* at 4 °C. In all, 15–50 μg of protein lysates, determined by BCA assay (Invitrogen-ThermoFisher Scientific) was denatured and separated by electrophoresis using 8–16% Tris-Glycine Mini Gels (Invitrogen-ThermoFisher Scientific) and then electro-blotted onto PVDF membranes (Bio-Rad Laboratories Inc., Hercules, CA). Membranes were blocked with TBS/0.1%-Tween20 (TTBS) containing 5% non-fat dry milk before overnight incubation with the specified antibodies. Peroxidase-conjugated anti-mouse and anti-rabbit secondary antibodies (Jackson ImmunoResearch, Laboratories Inc.) were added for 1 h at room temperature in the same buffer as used for the primary antibodies (2.5% non-fat dry milk in TTBS). Reactive bands were detected using Clarity Max^TM^ Western ECL Substrate (Bio-Rad Laboratories Inc.), according to the manufacturer’s instructions. Densitometry of Western blot bands was performed with the ImageJ software^58^. Primary antibodies used for Western blotting analysis were as follows: rabbit monoclonal anti-CLN5 [EPR12197(B)] (Abcam #ab170899; dilution 1:1000), mouse monoclonal anti-P62 (BD biosciences #610832; dilution 1:1000), rabbit monoclonal anti-LC3B (Sigma-Aldrich #L7543; dilution 1:1000), rabbit polyclonal anti-ACO2 (Bethyl laboratories #A305–302A-M; dilution 1:1000), rabbit polyclonal anti- ATP5L (Bethyl laboratories #A305–486A-M; dilution 1:500), mouse monoclonal anti-FUNDC (Santa Cruz Biotechnology #sc-517152; dilution 1:500). Immunodetection with mouse monoclonal anti-GAPDH antibody [6C5] (Abcam # ab8245; dilution 1:8000) served as a loading control to normalize the bands intensity.

### Statistical analyses

Data presentation and statistical analyses were performed using Prism 8 (GraphPad Software, San Diego CA). For functional studies and Western blot analysis, data were presented, stating n as a mean ± standard deviation (SD) from at least three independent experiments. Statistical analyses utilized Student *t* test with significance set at *P* < 0.05 (*), *P* < 0.01 (**), and *P* < 0.001 (***). For proteome analysis, six different mitochondrial fractions from two distinct *CLN5* KO genotypes were compared with three mitochondrial samples obtained from a Ctrl cell line. Each biological replicate was analyzed in technical triplicates. To compare the controls and KO lines, we utilized the between-subject design scheme of the Progenesis QI^TM^ software (Nonlinear Dynamics, Durham, NC). The ANOVA calculation applied by this scheme assumes that the conditions are independent and used a statistical test which presumes that means of the conditions are equal and the variance similar between the groups. *P* values ≤ 0.05 by ANOVA were considered as significant.

## Supplementary information

revised legend to supplem figures

Supplementary Fig. S1

Supplementary Fig. S2

Supplementary Fig. S3

Supplementary Table S1

Supplementary Table S2

Supplementary Table S3

Supplementary Table S4

checklist

declaration contribution authors

## Data Availability

All data generated or analyzed during this study are included in this published article and its Supplementary Information files. Mass spectrometry data have been deposited in the Mass Spectrometry Interactive Virtual Environment (MassIVE) database under accession number MSV000084880.
