# Supplementary material for: Proteomic and functional analyses in disease models reveal CLN5 protein involvement in mitochondrial dysfunction
Source: Cell Death Discov. 2020 Mar 30;6:18. doi: 10.1038/s41420-020-0250-y (PMC7105465; doi:10.1038/s41420-020-0250-y)
Supplement: Supplementary file 1 — revised legend to supplem figures [file 41420_2020_250_MOESM1_ESM.docx]

**Supplementary figure legends**

**Fig. S1** Molecular profiles and genotype-phenotype correlation of used cell models. **a.**The editing efficiency in HEK 293T and SH-SY5Y clones, selected for downstream analyses, was confirmed by direct Sanger sequencing, Electropherograms show random indel events in correspondence with the sequence of the functional sgRNA, resulting in a premature stop codon. **b.** CLN5 protein levels, measured by Western blotting, show a full lacked CLN5-immunoreactivity in KO cell lines. We intentionally reported for SH-SY5Y cells the same image shown in Figure 2A. **c**. Immunochemical studies reveal intracellular accumulation of subunit c of mitochondrial ATP synthase (SCMAS) in SH-SY5Y KO cells. Scale bar, 10 µm.

**Fig. S2** Network analysis in *CLN5* KO cells**. a** Interaction network annotating statistically relevant disease function and canonical pathways with the lowest predicted score to mtDEPs with in HEK 293T KO model. **b** Identification of common altered cellular pathways by a bioinformatic scrutiny of mtDEP applying Gene Ontology “biological process” criteria. Pathways with the highest predicted -log_10_ (p value) are reported. The number of mitochondrial DEPs assigned to each category is reported.

**Fig. S3** Network analysis in *Cln5^-/-^* mouse. **a** Interaction network annotating statistically relevant disease function and canonical pathways with the lowest predicted score to mtDEPs with in *Cln5 ^-/-^* model. **b** Identification of common altered pathways in mouse model by a bioinformatic scrutiny of mtDEP applying Gene Ontology “biological process” criteria. Pathways with the highest predicted -log_10_ (p value) values are reported. The number of mitochondrial DEPs assigned to each category is presented.
