# Supplementary figures and images for: Proteomic and functional analyses in disease models reveal CLN5 protein involvement in mitochondrial dysfunction
Source: Cell Death Discov. 2020 Mar 30;6:18. doi: 10.1038/s41420-020-0250-y (PMC7105465; doi:10.1038/s41420-020-0250-y)

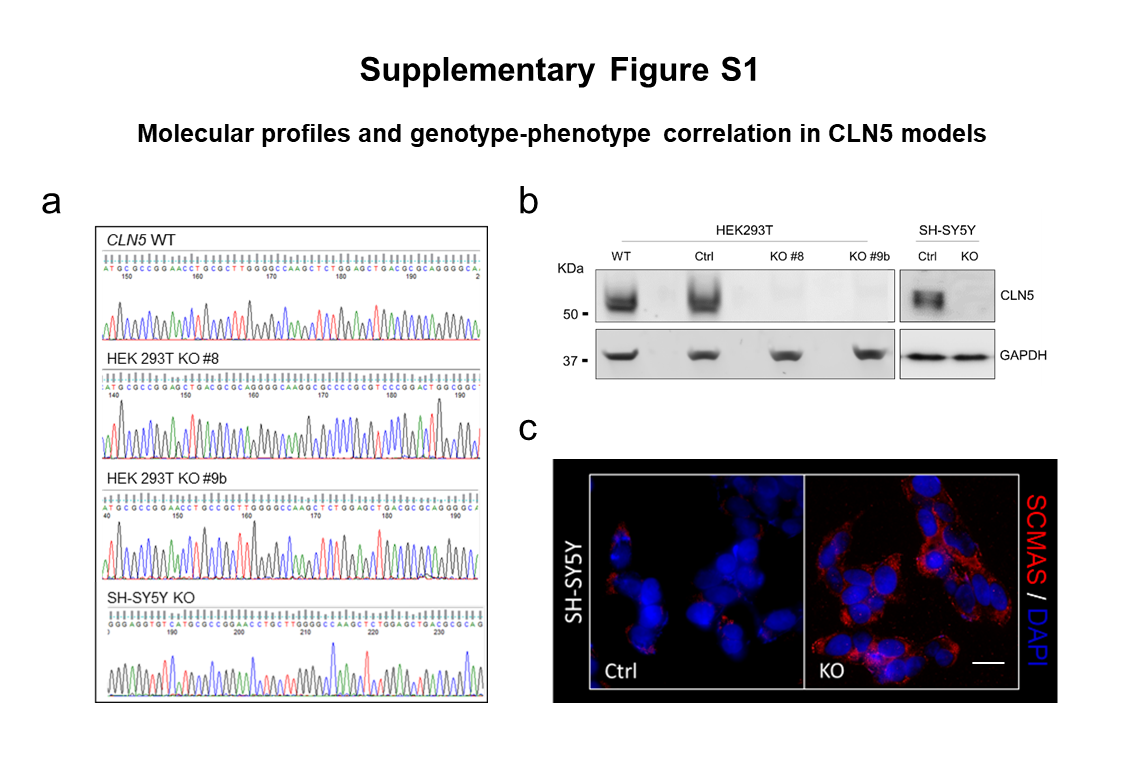

Supplement: Supplementary file 2 — Supplementary Fig. S1 [file 41420_2020_250_MOESM2_ESM.tif]

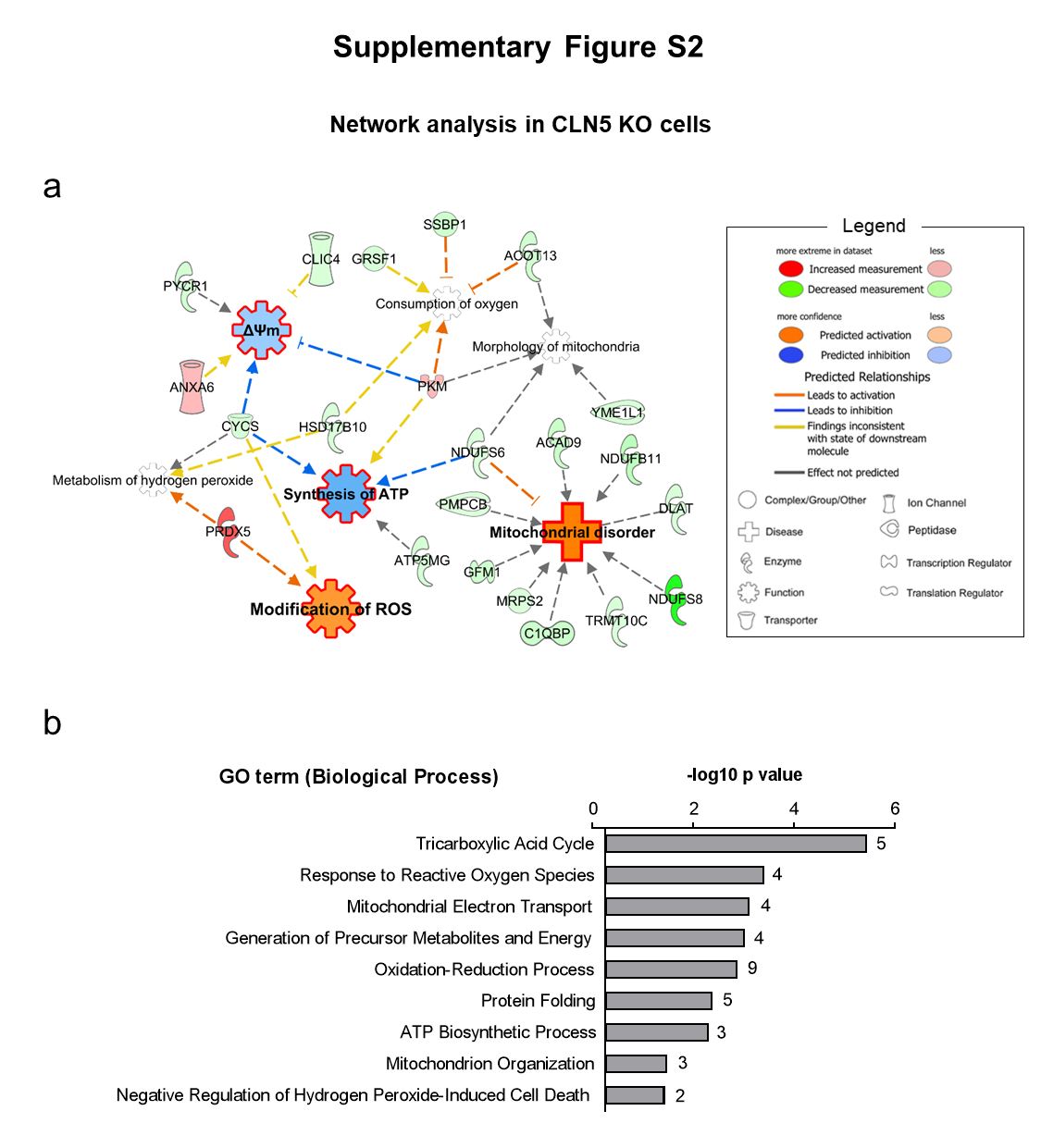

Supplement: Supplementary file 3 — Supplementary Fig. S2 [file 41420_2020_250_MOESM3_ESM.tif]

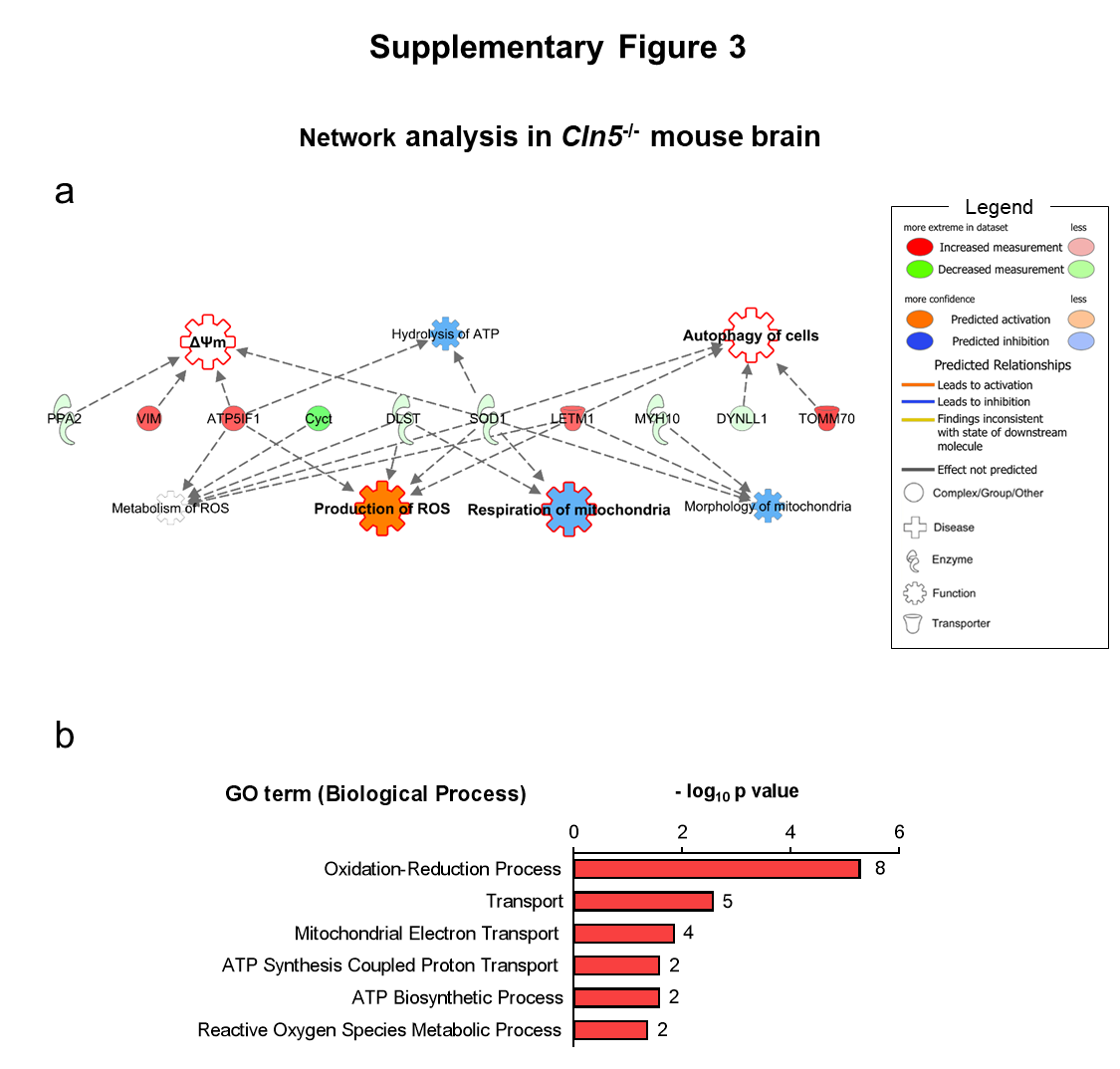

Supplement: Supplementary file 4 — Supplementary Fig. S3 [file 41420_2020_250_MOESM4_ESM.tif]
