# Supplementary material for: Proteomic and functional analyses in disease models reveal CLN5 protein involvement in mitochondrial dysfunction
Source: Cell Death Discov. 2020 Mar 30;6:18. doi: 10.1038/s41420-020-0250-y (PMC7105465; doi:10.1038/s41420-020-0250-y)
Supplement: Supplementary file 10 — declaration contribution authors [file 41420_2020_250_MOESM10_ESM.pdf]

DECLARATION OF CONTRIBUTIONS TO ARTICLE **ADMC**

Manuscript Number: CDDISCOVERY-20-0841-T

Journal Name: *Cell Death Discovery* (the 'Journal')

Proposed Title of the Contribution:

Proteomic and functional analyses in disease models reveal CLN5 protein involvement in mitochondrial dysfunction (the 'Contribution)

Author(s):

Stefano Doccini, Federica Morani, Claudia Nesti, Francesco Pezzini, Giulio Calza, Rabah Soliymani, Giovanni Signore, Silvia Rocchiccioli, Katja M. Kanninen, Mikko T. Huuskonen, Marc Baumann, Alessandro Simonati, Maciej Lalowski, Filippo M. Santorelli (the 'Authors')

For all *CDDis*covery articles, each person named as an author in the published version must be able to show he or she has contributed substantially to the article.

Authorship credit should be based on 1) substantial contributions to conception and design, acquisition of data, or analysis and interpretation of data; 2) drafting the article or revising it critically for important intellectual content; and 3) final approval of the version to be published. Authors should meet conditions 1, 2 and 3.

Any person who cannot be shown to have made a substantial contribution to the article cannot be listed as an author in the final version. The name of any person who is deemed to have made a minor contribution can, however, appear in the Acknowledgments section of the article.

Please complete the table below to indicate the contributions of all named authors to the manuscript.

Author Full Name:	Specification of Contribution to the Manuscript:
Stefano Doccini	conception and design, acquisition of data, analysis and interpretation of data; drafting the article, revising it critically for important intellectual content; final approval of the paper
Federica Morani	acquisition of data, analysis and interpretation of data; revising the article critically for important intellectual content; final approval of the paper
Claudia Nesti	acquisition of data, analysis and interpretation of data; revising the article critically for important intellectual content; final approval of the paper
Francesco Pezzini	acquisition of data, analysis and interpretation of data; revising the article; final approval of the paper
Giulio Calza	acquisition of data, analysis and interpretation of data; revising the article; final approval of the paper
Rabah Soliymani	acquisition of data, analysis and interpretation of data; revising the article; final approval of the paper
Giovanni Signore	acquisition of data, analysis and interpretation of data; revising the article; final approval of the paper
Silvia Rocchiccioli	acquisition of data, analysis and interpretation of data; revising the article; final approval of the paper
Katja M. Kanninen	analysis and interpretation of data; revising the article; final approval of the paper
Mikko T. Huuskonen	analysis and interpretation of data; revising the article; final approval of the paper
Marc Baumann	analysis and interpretation of data; revising the article; final approval of the paper
Alessandro Simonati, Maciej Lalowski, FM Santorelli	conception and design, interpretation of data; revising the article for important intellectual content; final approval of the paper

Please complete the table below to indicate the contributions of all named authors to the figures.

Figure 1:

Contribution: all

Figure 2:

Contribution: all

Figure 3:

Contribution: all

Figure 4:

Contribution: all

Figure 5:

Contribution: all

Figure 6:

Contribution: all

Signed for and on behalf of the Author(s):	Print Name:	Date:
[signature]	FILIPPO M. SANTORELLI	23-01-2020
